# Negative pressure technology enhances bone regeneration in rabbit skull defects

**DOI:** 10.1186/1471-2474-14-76

**Published:** 2013-03-03

**Authors:** Yin-gang Zhang, Zhi Yang, Hong Zhang, Miao Liu, Yushen Qiu, Xiong Guo

**Affiliations:** 1 Department of Orthopedics, First Affiliated Hospital of Medical College of Xi’an Jiaotong University, Xi’an 710061, China; 2 Key Laboratory of Environment and Genes Related to Diseases, Ministry of Education, Xi’an Jiaotong University, Xi’an 10061, China

**Keywords:** Negative pressure, Bone regeneration, VEGF, BMP-2, Animal experimental use

## Abstract

**Background:**

Bone is a slowly regenerating tissue influenced by various physiological processes, including proliferation, differentiation, and angiogenesis, under the control of growth factors. Shortening this healing time is an important and popular clinical research focus in orthopedics. Negative pressure can stimulate angiogenesis, improve blood circulation, promote granulation tissue growth and accelerate tissue wound healing. We sought to determine whether negative pressure could reduce bone healing time in a rabbit cranial defect model.

**Methods:**

Four symmetrical holes (diameter, 3.5 mm) were drilled into the skulls of 42 New Zealand white rabbits, with two holes in each parietal bone. For each rabbit, the two sides were then randomly assigned into experimental and control groups. Using negative pressure suction tubes, experimental holes were treated with −50 kPa for 15 minutes, four times per day, whereas the control holes remained untreated. After 4 weeks, the negative pressure suction tubes were removed. At 2, 4, 6 and 8 weeks, three-dimensional (3D) reconstruction computed tomography (CT), X-ray radiopacity, and two-photon absorptiometry were used to evaluate new bone formation. Histological changes were determined by hematoxylin and eosin (H.E) staining. At weekly intervals until 6 weeks, the mRNA expression levels of vascular endothelial growth factor (VEGF) and bone morphogenetic protein (BMP)-2 were evaluated by RT-PCR. A paired student’s *t*-test was employed to compare X-ray radiopacity and bone density measurements between the experimental and control groups.

**Results:**

3D-reconstruction CT showed that new bone regeneration in the experimental group was greater than that in the control group at 4 and 6 weeks. At these time points, the experimental group presented with higher X-ray radiopacity and increased bone density (*P* < 0.05) as compared with the control group. Cartilage islands and new bone were observed by H.E staining at 2 weeks in the experimental group. By 6 weeks, the new bone had matured into lamellar bone in the experimental group. RT-PCR results showed that VEGF and BMP-2 were highly expressed in the experimental group as compared with control.

**Conclusions:**

Intermittent negative pressure can promote the regeneration of bone possibly by enhancing the expression of VEGF and BMP-2.

## Background

Vacuum-assisted closure (VAC) was first proposed in 1993 by Fleischmann and colleagues for the treatment of open or infected wounds [1]. By 1997, following improvements in the design, VAC was used for the treatment of complex, chronic wounds. The therapeutic effect of VAC in chronic wound repair has gained wide attention over the past decade, and the list of diseases that can be treated by VAC is continuously expanding. Currently, VAC has been shown to play an important role in the treatment of limb wounds, soft tissue defects, chronic osteomyelitis, osteofascial compartment syndrome, amputation and replantation [2]. Also known as negative-pressure wound therapy (NPWT), VAC can stimulate angiogenesis, improve blood circulation, promote cell growth of granulation tissue and accelerate wound healing processes through its mechanical and biological effects on the soft tissue [3].

Bone repair is a slow process affected by many factors, including cell proliferation, differentiation, angiogenesis and other biological and mechanical factors [4]. How to shorten bone healing time and reduce the incidence of nonunion are two popular research focuses for orthopedic clinicians and researchers. In bone tissue regeneration and remodeling, hemodynamic factors and stress stimuli are critical factors that accelerate bone formation. Negative pressure achieves its regenerative effect on the soft tissues by affecting these hemodynamic factors and stress stimuli. Previously, we investigated the effect of intermittent negative pressure on the proliferation and differentiation of bone marrow mesenchymal stem cells (BMSCs) into cells that expressed markers characteristic of osteoblasts. These results showed that intermittent negative pressure could promote osteogenic differentiation of BMSCs, upregulate the expression of bone-related genes and improve the osteogenic activity of these cells. These studies thus established a cytological basis for the application of negative pressure technology in bone tissue repair [5-7]. In this study, we investigated the impact of negative pressure on the bone repair process in a rabbit skull defect model. We also determined the effect of negative pressure on the mRNA expression of vascular endothelial growth factor (VEGF) and bone morphogenetic protein (BMP)-2, which may provide more useful information for understanding the potential mechanism by which negative pressure influences bone formation.

## Methods

### Preparation of animal model and grouping

A total of 42 New Zealand white rabbits (age: 2–2.5 months; body weight: 1.5-2.0 kg; gender: male and female) were provided by Xi’an Jiaotong University School of Medicine Animal Experimental Center. Ketamine hydrochloride (0.5 ml/kg) and Sumianxin (1 ml/kg) were used for rabbit anesthesia. Skin preparation, disinfection, draping, incisions in the skin, and subcutaneous tissue, deep fascia and periosteal stripping were performed according to standard protocols. Four symmetrical holes, each with a diameter of 3.5 mm, were drilled into the parietal bones of each rabbit skull through all the layers to the dura matter with 2 holes in each bone using a 3.5 mm power drill; this method could avoid injury to the soft tissues as the drill penetrated the bone. Vacuum suction tubes were then placed onto the surfaces of the experimental holes. Following this, the soft tissue wound was closed, and the tubes were subcutaneously led out. In order to prevent having to change the tubes while the rabbits were mobile, hard braces were used to protect the tops of the rabbit skulls.

**Figure 1:**
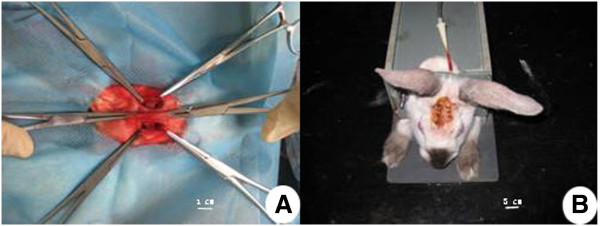
**Preparation of animal model.** (**A**) Preparation of skull defect. For each rabbit, two 3.5 mm holes were drilled on each side of skull, respectively. The two holes on one side of skull were used as control group, and the two holes on the other side of skull were used as experimental group in each rabbit. (**B**) Postoperative outline of negative vacuum tube. The vacuum tube was placed near two experimental holes. For each rabbit, −50 kPa of negative pressure was applied to two experimental holes for 15 min, which was performed 4 times per day.

There was no postoperative infection, but 2 rabbits died due to diarrhea. After the rabbits had recovered from anesthesia, the negative pressure was applied (Figure 1). The vacuum suction tube was connected to a micro-vacuum instrument (Germany Berenger), and negative pressure was applied at −50 kPa for 15 minutes, four times per day. After 4 weeks, the vacuum suction tubes were removed under anesthesia. Postoperative penicillin at a dose of 800,000 U was injected intramuscularly for 3 days. All animal procedures were performed in accordance with institutional guidelines (Animal Care and Veterinary Services, Xi’an Jiaotong University, ID: 09-A01).

**Figure 2:**
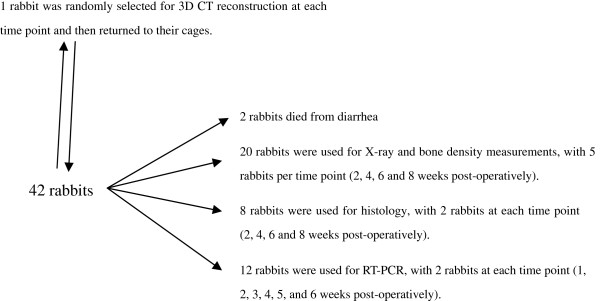
**Experimental animals.** A total of 42 rabbits were used in this study.

Animals were randomly grouped for the various tests. Figure 2 illustrates the assignment of rabbits for the various tests. Before the operation, the staff not directly participating in the surgery drew lots randomly to decide which side would be the experimental group for each rabbit. The surgeons were informed of these group assignments after the bone defects had been created. A blinded principle was adopted to estimate the growth of bone postoperatively, with the observer and data processer blinded to the side groupings in each rabbit.

### 3D CT reconstruction

At 2, 4, 6 and 8 weeks after surgery, one rabbit was selected at random to perform 3D CT reconstruction under anesthesia, using cranial CT coronal TLC scanning, with a slice thickness of 0.75 mm. The screening raw data were imported into the SIEMENS workstation and a 3D coronal reconstruction was implemented using the Syngo software (Siemens Medical Solutions, Erlangen, Germany). Following recovery from anesthesia, the rabbits were returned to their cages for the duration of the experiment.

### X-ray examination and bone density examination

Five rabbits were sacrificed at 2, 4, 6 and 8 weeks, respectively. Intact parietal bones were harvested and examined by X-ray (1.2 s, 50 KV, 5.4 mA, 50 cm projection). X-ray radiopacity for the parietal bones in each group was calculated from the X-ray images using Photoshop CS 8.01 (Adobe Systems, San Jose, CA, USA). Differences between the experimental and control parietal bones was also compared based on the degree of radiopacity, calculated with the following equation: radiopacity degree = bone radiopacity area/total hole area × 100%. For the variations of hole areas of a rabbit, there was no statistically significant difference between the experimental groups. Following X-ray scanning, the harvested parietal bones were then examined using two-photon absorptiometry (model DCS600, Aloka Co., Tokyo, Japan) to assess the changes in bone density between the experimental and control holes in each rabbit parietal bone.

### Histological examination

Two rabbits were sacrificed at 2, 4, 6 and 8 weeks post-surgery for histological analyses. The parietal bones from each rabbit were harvested and subjected to conventional decalcification, embedding, sectioning and H.E staining. The histological features of the new bones were examined by light microscopy (Sichangyue Optical Instrument Co., Ltd., Shanghai, China).

### Real-time PCR

Two rabbits were sacrificed at weekly intervals until 6 weeks post-surgery for analysis of the mRNA expression changes in VEGF and BMP-2 and the parietal bones were harvested from each rabbit. Because of the limited RNA that was obtained from the tissue in each hole, the tissues from a total of four holes were pooled to obtain one sample for the experimental group and one for the control. The tissue was then snap-frozen in liquid nitrogen and ground into a powder using a mortar and pestle. The liquid nitrogen was allowed to evaporate, and total RNA was extracted using Trizol reagent. The concentration and purity of the extracted total RNA was measured using an ultraviolet spectrophotometer. Total RNA (2 μg) was reverse transcribed into cDNA using reverse transcriptase kit (DNA Technology, Aarhus, Denmark), and then amplified using real-time quantitative PCR under the following conditions: denaturing at 94°C for 30 s, annealing at 53°C for 30 s, and extension at 72°C for 30 s, for a total of 50 amplification cycles. The Ct values were calculated using the real-time quantitative PCR analysis program, and the ΔΔCt values are equal to Ct objective gene – Ct β-actin; the relative value of the objective gene was equal to 2-ΔΔCt value. The primer sequences were as follows: VEGF, (forward) 5^′^-GGAGTACCCTGATGAGATCGA-3^′^, (reverse) 5^′^-CTTTGGTCTGCATTCACATTTGT-3^′^; BMP-2, (forward) 5^′^-CGTGAGGATTAGCAGGTCTTTG-3^′^, (reverse) 5^′^-TTTCGCTTGACGCTTTTCTC-3^′^; β-actin (forward) 5^′^-AGGCACCAGGGCGTGAT-3^′^, (reverse) 5^′^-TCGTCCCAGTTGGTGACGAT-3^′^.

### Statistical analysis

The degree of X-ray radiopacity and changes in the bone density were expressed as the mean ± standard deviation. A two-sided paired student’s *t* test was conducted using SPSS17.0 software (SPSS, Chicago, IL, USA). A *P* value < 0.05 was considered statistically significant.

## Results

### Comparison of bone regeneration between experimental and control groups using 3D reconstruction CT

**Figure 3:**
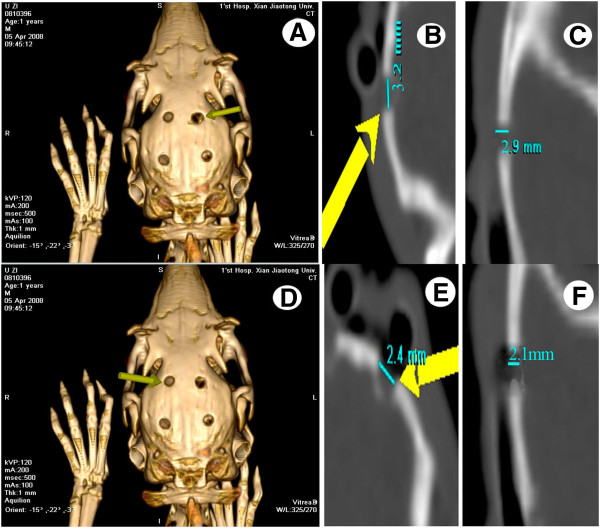
**Three-dimensional computed tomography (3D CT) reconstruction of rabbit parietal bones of control (A, B, C) and experimental (D, E, F) groups at 4 weeks after surgery. A** and **D**, images of 3 D CT reconstruction of rabbit parietal bone; **B** and **E** present the width of hole of rabbit parietal bone; **C** and **F** present the depth of hole of rabbit parietal bone. The hole of rabbit parietal bone in experimental group were smaller than that in control group.

At 2 weeks post-operatively, no significant changes in the skull defects were observed between the experimental and control groups. The defects remained at the same width and depth as compared with that at immediately after surgery (0 weeks). After 4 weeks, new bone appeared and the defects in the skulls had partially healed, with a reduced width and depth observed for all of the defects in the experimental group (Figure 3). In contrast, in the control group, the edges of the skull defects were vague, the centers of the defects were still clearly visible, and the widths and depths of the defects did not change significantly (Figure 3). Six weeks post-operatively, the skull defects in the experimental group had healed and the bone density in the healed bone was not significantly different to that in the surrounding uninjured bone. By comparison, the skull defects in the control group were repaired, but the density of the new bones was uneven, and different to that of surrounding bone tissues. At 8 weeks, all skull defects were fully restored, with no significant differences between repaired bone tissues and the surrounding bone tissues in either group.

### Comparison of X-ray radiopacity between the experimental and control groups

**Figure 4:**
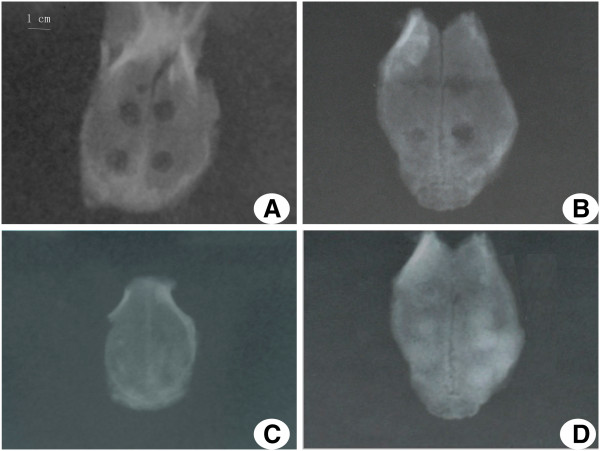
**X**-**ray imaging.** The experimental group (left) and the control group (right) at 4 weeks (**B**) and 6 weeks (**C**). The X-ray radiopacity in the experimental group was higher than that in the control group. **A** and **D** show representative radiopacity results at 2 weeks and 8 weeks, respectively.

**Figure 5:**
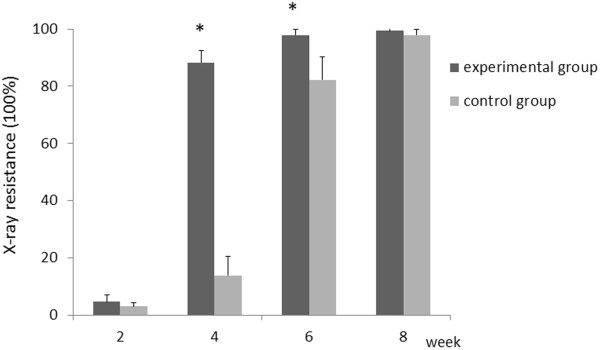
**X**-**ray radiopacity.** At 4 weeks and 6 weeks, the X-ray radiopacity of the bone formation in the experimental group was higher than that in the control group. At 2 and 8 weeks, there was no significant difference between the experimental and control groups. For each time point, the number of rabbits is 5 and the number of holes is 10. (N = 5, * denotes *P* <0.05).

The X-ray radiopacity in the experimental group was higher than that in the control group at 4 and 6 weeks post-operatively. The related images and data are shown in Figures 4 and 5, respectively. At 4 weeks, the X-ray radiopacity in the experimental group was 6-fold higher than that in the control group (88.28 ± 4.14 vs. 13.81 ± 6.74,t = 18.721, v = 9, *P* < 0.0005). At 6 weeks, the X-ray radiopacity in the experimental group was 18% higher than that in the control group (97.86 ± 2.11 vs. 82.27 ± 8.02, *t* = 4.925, v = 9, *P* = 0.004). In contrast, at 2 and 8 weeks after surgery, no significant differences in X-ray radiopacity were observed between the experimental and control groups (*P* > 0.05).

### Comparison of bone density between the experimental and control groups

**Figure 6:**
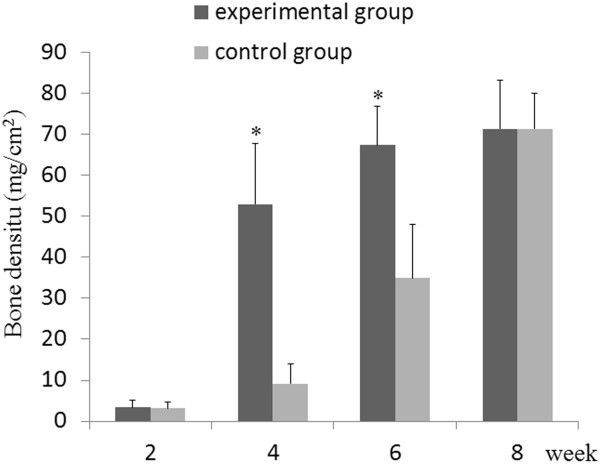
**Bone density of rabbit parietal bone at 2, 4, 6 and 8 weeks after surgery.** At 4 and 6 weeks after surgery, the bone density of rabbit parietal bone in experimental group was significantly higher than that in control group. At 2 and 8 weeks after surgery, there was no significant difference in bone density of rabbit parietal bone between experimental and control groups (* denotes P <0.05).

At 4 and 6 weeks post-surgery, the bone density in the experimental group was higher than that in the control group. At 4 weeks, the bone density was 6-fold higher in the experimental group than that in the control group (52.95 ± 14.89 vs. 9.17 ± 4.83, *t* = 6.766,v = 9, *P* = 0.001) and 2-fold higher at 6 weeks, respectively (67.38 ± 9.48 vs. 34.94 ± 13.10,*t* = 5.227,v = 9, *P* = 0.003). Similar to the X-ray radiopacity results, we observed no significant difference in bone density at 2 and 8 weeks, post-operatively between the two groups (*P* > 0.05) (Figure 6).

### Histological observations

**Figure 7:**
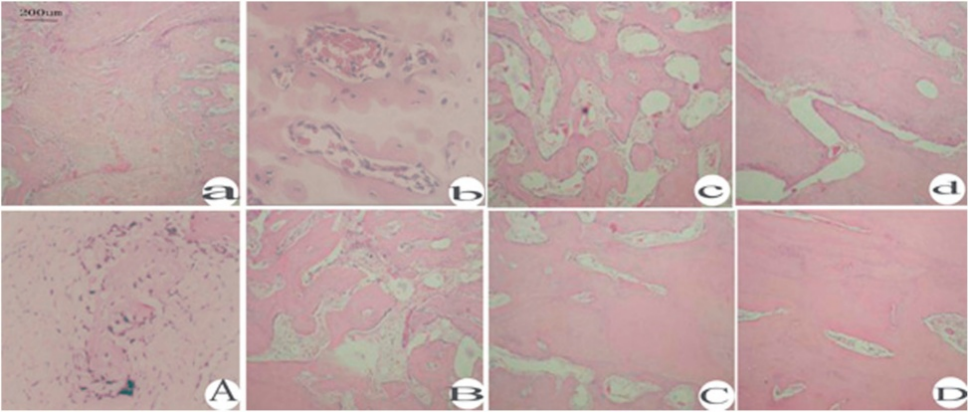
**H.E staining (magnification, x100).** At 2 weeks, the skull holes were filled with fibrous tissues in the control group (**a**), while cartilage islands and new bone formation was observed in the experimental group (**A**). At 4 weeks post-operatively, the trabecular bone in the control group (**b**) was small, sparse and mixed with cartilage islands and new bone formation, while in the experimental group (**B**), trabecular bone was relatively thick and dense. At 6 weeks post-operatively, the trabecular bone in the control group (**c**) became thick and dense, while, the trabecular bone in the experimental group (**C**) became lamellar bone. At 8 weeks postoperatively, lamellar bone was found in both groups (**D** and **d**).

H.E staining at 2 weeks post-operatively showed that the majority of the bone defects in the control side of the rabbit parietal bones were filled with fibrous tissues, while cartilage islands and the new bone were observed in the experimental side of the rabbit. At 4 weeks, the proportion of trabecular bone in the control group was small, sparse and mixed with islands of cartilage formation and new bone. In comparison, the trabecular bone in the experimental group was relatively thick and dense. At 6 weeks, the trabecular bone in the control group appeared thick and dense, while the trabecular bone in the experimental group had become lamellar bone. By 8 weeks, the holes in the parietal bones of both sides of the rabbit were filled with lamellar bone (Figure 7).

### Expression of VEGF and BMP-2 were increased in the experimental group

**Figure 8:**
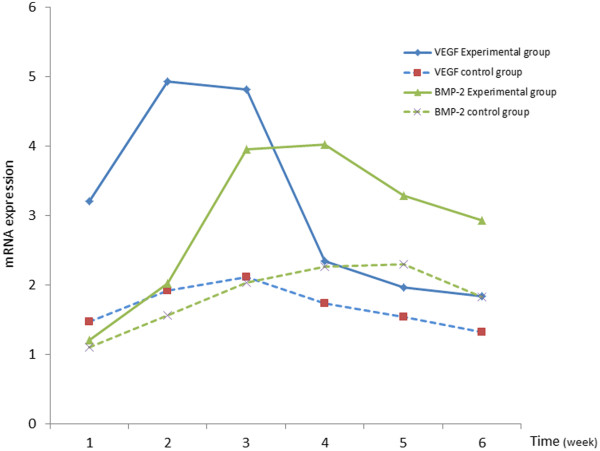
**Expression of VEGF and BMP-2.** VEGF and BMP-2 mRNA expression in the experimental group was higher than that in the control group at each of the six time points examined.

RT-PCR analysis showed that the expression level of VEGF gene in the experimental group was significantly higher than that in the control group. At 2 weeks, the expression level of VEGF in the experimental group was 2-fold higher than that in the control group (4.936 vs. 1.921). In the control group, the expression of VEGF was maximal at 2–3 weeks post-surgery and decreased at 4–6 weeks post-surgery. In the experimental group, the expression curve was similar to the control group. However, the expression level of VEGF at 2–3 weeks in the experimental group was obviously higher than that in the control group. Additionally, the mRNA expression level of BMP-2 in the experimental group was higher than that in the control group. At 3 weeks post-surgery, the BMP-2 mRNA level in the experimental group was 2-fold higher than that in the control group (3.956 vs. 2.031). In the control group, the expression level of BMP-2 reached a maximum level at 3–4 weeks post-operatively, and decreased at 5–6 weeks post-operatively In the experimental group, the expression level of BMP-2 reached a maximum at 3–4 weeks post-operatively, and the level was obviously higher than that in the control group (Figure 8).

## Discussion

Previous studies have shown that bone formation depends on three osteogenic factors [8]: 1) a certain number of bone marrow mesenchymal stem cells; 2) effective inducing factors; and 3) the presence of a micro-environment permissive for new bone formation. The activation of internal or external osteogenic factors represents two feasible approaches to facilitate the regeneration and repair process in bone. Exogenous osteogenic factors mainly included stem cells, growth factors and gene therapies [9,10]. However, these approaches have a high associated cost and a certain degree of technical difficulty. In addition, the clinical efficacy and safety of these approaches still need to be addressed. Traditional approaches for enhancing internal osteogenic factors to accelerate the fracture healing process focus on physical therapies, such as electromagnetic field therapy [11], high-energy shock waves [12] and low-intensity pulsed ultrasound [13]. However, these methods are not common in clinical practice owing to their complex operation, dependence on large equipment, and uncertain efficacy. Therefore, developing a simple, economic, safe and reliable approach to promote bone repair is still an important task of orthopedic research.

Vacuum-assisted closure (VAC) can stimulate angiogenesis, improve wound blood supply, enhance the growth of cells and the formation of granulation tissue, and accelerate soft tissue wound healing processes. VAC therefore plays an important role in the treatment of soft tissue injury. Because hemodynamic factors and stress stimuli are critical for bone tissue regeneration and remodeling, and negative pressure achieves its effect by affecting these factors, we speculated that negative pressure technology could enhance the expression of endogenous osteogenic factors and promote bone regeneration. Thus, the purpose of this study was to investigate the role of intermittent negative pressure on bone tissue regeneration.

This study was designed according to several preliminary tests. First, for the ease of making comparisons and to shorten the testing time, we did not use the conventional bone defect diameter of a critical-sized defect. Instead, we adopted a smaller diameter of 3.5 mm because in our pre-preliminary experiments, we found that at 6 weeks post-operatively, the defects in the skulls of rabbits were filled with regenerated bone in both the experimental and control groups. We used a 3.5 mm drill, which to the naked eye, showed no obvious disturbances to the dura and the underlying tissues after drilling. However, we did not study this in detail, and future studies may determine the effects, if any, of drilling on the dura and the underlying tissues. Second, we determined the changes in VEGF and BMP-2 gene expression at weekly intervals over a 6-week timeframe in order to identify early subtle changes in gene expression; by comparison, other tests were conducted at 2, 4, 6 and 8 weeks. Third, previous cell culture studies indicated that a pressure of −50 kPa could induce differentiation of human mesenchymal stem cells into bone cells, promote osteoprotegerin (OPG) mRNA expression, and reduce OPG ligand (OPGL) mRNA expression [5]. Therefore, in this experiment, we applied −50 kPa for 15 minutes, 4 times per day over a 4 week time frame. Since the fracture repair process is divided into four stages – inflammation (1-7 days), soft callus formation (2-3 weeks), hard callus formation, and remodeling – we deemed that the first and second stages would show the most remarkable changes to cellular metabolism and growth factor expression. Therefore, we chose a four-week timeframe for its administration. Fourth, since infection caused by placement of a suction tube for four weeks may complicate our results, we used conventional antibiotics postoperatively. These limitations in the study design can only explain a superficial mechanism of the effects of negative pressure and, thus, further studies are needed to gain a more detailed understanding of the molecular mechanism of negative pressure-induced bone regeneration. A further limitation in this study was the absence of a ‘0 week’ time point, where the animals were sacrificed immediately following surgery to mark the original defect size. Moreover, there would be many difficulties for negative pressure technique in clinical application, such as how to get to the site of fracture, how to keep the negative pressure at the fracture site, infection in vivo et al. These will also be included in future work. However, despite these limitations, we identified an obvious effect of negative pressure in this rabbit defect model as compared with the untreated control.

The 3D reconstruction CT scans showed a reduction in the size of the skull defects in the experimental group at 4 weeks, their bony integration by 6 weeks, and complete healing by 8 weeks. Complete healing was also seen in the control group at 8 weeks, indicating that 3.5 mm-sized defects can heal naturally. X-ray radiopacity and bone density in the experimental group was higher than those in the control group at 4 and 6 weeks after surgery, but no differences were observed between the two groups at 2 and 8 weeks post-surgery, consistent with the CT scanning results. Histological examination showed the appearance of woven bone at 4 weeks and lamellar bone at 6 weeks in the experimental group, whereas woven and lamellar bones appeared at 6 and 8 weeks, respectively, in the control group. Woven-like new bone appears in the middle stages of skull repair, while lamellar structure appears in the later stages. Thus, the accelerated skull defect healing after negative pressure supports our previous findings that intermittent negative pressure can promote bone tissue regeneration.

RT-PCR results showed that, in the fracture healing process, negative pressure can significantly increase VEGF gene expression, which peaked at 2–3 weeks post-operatively. These changes appeared earlier than the other more general changes to the bone injury site, as was determined by CT scanning, X-ray and bone density measurements. VEGF-mediated angiogenesis is a basic requirement for bone growth. Recent studies have shown that VEGF also plays an important role in the differentiation of osteoblasts and recruitment of osteoclasts [14,15]. The functional effect of VEGF on osteoblasts can be achieved via three ways. First, VEGF couples angiogenesis and osteogenesis. Second, VEGF can promote osteoblast differentiation and improve its osteogenic activity. Third, VEGR can promote neighboring cells to secrete a variety of cytokines, which, in turn, enhance the activity of osteoblasts [16,17]. Deckers et al., [17] found that in the early stages of osteoblast differentiation, VEGF was expressed at a low level. However, during the final stages of osteoblast differentiation, its expression level was significantly increased and reached a peak expression during tissue mineralization. We also observed that, during the process of fracture healing, negative pressure can also promote the expression of BMP-2, which reached a maximum expression at 3–4 weeks post-operatively. BMPs are a family of multi-functional cytokines belonging to the transforming growth factor (TGF) family. BMP-2, BMP-4 and BMP-6 supplementation to osteoblasts results in cell mineralization and the increased secretion of VEGF [18]. Recombinant human BMP-2 can promote the differentiation of MSCs to chondrocytes and osteoblasts.

Together, an increased expression of VEGF creates a favorable vascular environment for bone tissue growth [18]. BMP-2 then not only increases the expression of VEGF, but also increases the synthesis and secretion of VEGF-B and VEGF-C [19]. Overall, during bone repair, the function of VEGF and BMP are complementary. On one hand, although BMP cannot directly induce angiogenesis, it can promote angiogenesis and accelerate the bone tissue healing process by stimulating VEGF expression. On the other hand, VEGF promotes the proliferation and differentiation of chondrocytes, osteoblasts and osteoclasts, and up-regulates the expression level of BMP, which can promote the differentiation of MSCs to chondrocytes and osteoblasts differentiation. Based on the results of previous studies and studies from our laboratory, it is reasonable to speculate that negative pressure on the rabbit skull repair might partly induce the elevation of VEGF and BMP-2 expression levels, and the synergistic effect of these two growth factors stimulates angiogenesis, thereby increasing the blood supply to the wound and increasing the capacity for bone formation.

## Conclusions

Our study shows that intermittent negative pressure can promote bone regeneration possibly by increasing expression of VEGF and BMP-2. These results suggest that intermittent negative pressure is an effective approach for promoting bone formation.

## Competing interests

All authors have no financial and personal relationships with the organization that could inappropriately influence (bias) the current research. The authors declare that they have no commercial interests and no conflicts of interest in this study.

## Authors’ contributions

ZYG made substantial contributions to the conception and design, acquisition of data, analysis and interpretation of data, and drafting of the manuscript; YZ carried out the animal model and the RT-PCR analysis; ZH participated in the design of the study and performed the statistical analysis. LM, GX and QYS participated in the design and coordination of the study, and assisted with drafting the manuscript. All authors carried out the analyses, read, and approved the final manuscript.

## Pre-publication history

The pre-publication history for this paper can be accessed here:

http://www.biomedcentral.com/1471-2474/14/76/prepub
